# A New Departure at St. George's Infirmary

**Published:** 1913-01-11

**Authors:** 


### A NEW DEPARTURE AT ST. GEORGE'S INFIRMARY.

The very first entertainment in the history of St. George's Union Infirmary, Fulham Road, S.W., was given by some of the nursing staff to their colleagues and friends on Monday, December 30, at 7.30 p.m. The programme, which was in two parts, consisted of songs, duets, and choruses, and each part closed with a short farce acted with much spirit by the staff. As no men were allowed to take part in the programme, the male parts were also taken by sisters, whose unwonted attire caused much amusement. The music was rendered by the members of the Cow-girl Choir, who were all appropriately attired in red blouses, brown skirts, and fringed mocassins, and wore large floppy straw-hats turned up at the side cow-boy fashion. The dresses were designed, cut-out, and made by members of the nursing staff. On Thursday and Friday last week the staff had their Christmas dinners, which were attended by the guests in fancy dress, as the dinners were to be followed by small and early private dances. The tables looked very queer with so many strange figures round them. The dresses were mostly home-made, and at a cost not exceeding 2s. 6d., and where so many were remarkable it does not do to particularise. The prizes for the most beautiful dresses went to Sister Swayne and Nurse Mackenzie; for the most original to Nurse Dall and Nurse Fuller. The festal season has been most happy, and the greatest thanks are due to those who laboured so hard to make everything successful, and to the broad-minded Matron, who gave them the encouragement of her presence and counsel.

